# Drug and Neurofilament Levels in Serum and Breastmilk of Women With Multiple Sclerosis Exposed to Natalizumab During Pregnancy and Lactation

**DOI:** 10.3389/fimmu.2021.715195

**Published:** 2021-08-26

**Authors:** Undine Proschmann, Rocco Haase, Hernan Inojosa, Katja Akgün, Tjalf Ziemssen

**Affiliations:** Multiple Sclerosis Center, Center of Clinical Neuroscience, Department of Neurology, University Hospital Carl Gustav Carus, Dresden University of Technology, Dresden, Germany

**Keywords:** multiple sclerosis, natalizumab, pregnancy, lactation, neurofilament light chain, natalizumab concentration, breastmilk

## Abstract

**Objective:**

To determine the transfer of the monoclonal antibody natalizumab into breastmilk and to evaluate drug and serum neurofilament light chain ((s)NfL) levels in natalizumab exposed pregnancies and lactation periods.

**Methods:**

Eleven women with relapsing remitting multiple sclerosis treated with natalizumab during pregnancy and lactation were included in this study. Breastmilk samples were collected up to 302 days after delivery and analyzed for natalizumab concentration and NfL. Additionally, maternal drug levels and sNfL were determined preconceptually, in each trimester, at delivery and postpartum. Clinical and radiological disease activity was systemically assessed across pregnancy and postpartum period.

**Results:**

The mean average natalizumab concentration in breast milk was low at 0.06 µg/ml [standard deviation (SD) 0.05] in the eight patients who provided serial breastmilk samples with an estimated mean absolute infant dose of 0.007 mg/kg/d (SD 0.005). The relative infant dose (RID), a metric comparing the infant with maternal drug exposure was low as well with a mean of 0.04% (SD=0.03). Most patients had a maximum concentration in breast milk at one to eight days after infusion. Pregnancy was associated with a non-significant decline of the median natalizumab serum concentration. All patients exposed to natalizumab prior (n=10) and during pregnancy (n=11) kept free of disease activity during gestation. While pregnancy was associated with low sNfL levels in patients treated with natalizumab prior and during pregnancy, the postpartum period was linked to a transient sNfL increase in some patients without any evidence of clinical or radiological disease activity. NfL was detectable in the majority of breastmilk samples with a median concentration of 1.7 pg/ml (range 0.004-18.1).

**Conclusion:**

We determined transfer of natalizumab into breastmilk with an RID far below the threshold of concern of 10%. Studies including childhood development assessment are needed in order to gain safety data about natalizumab-exposed breastfeeding. SNfL assessment might be a useful adjunct to monitor silent disease activity and therapeutic response during pregnancy and postpartum period. However, further investigations regarding transient postpartum sNfL increases are required to determine its association to parturition per se or to a silent disease activity in people with multiple sclerosis.

## Introduction

Multiple sclerosis (MS) is a chronic autoimmune demyelinating disease of the central nervous system, which predominantly affects women, and is most commonly diagnosed in the early adulthood (1, 2). Thus, many people with MS (pwMS) will not have completed their family planning at the time of diagnosis. The natural reduction in relapses that occurs especially during the third trimester of pregnancy may not be sufficient to control disease activity, particularly in those pwMS with a highly active disease course (3, 4). Withdrawal of some of the more highly efficacious disease modifying drugs has been associated with a return or even rebound of disease activity during pregnancy (4–7).

Natalizumab (NAT; Tysabri^®^, Biogen, Cambridge, MA), as one of the highly efficacious treatments, can be continued in selected patients throughout pregnancy after weighting benefits for the mother against the potential risks to the fetus (8). NAT is a humanized anti-α4 integrin Immunoglobulin(Ig)G4 antibody that binds the α4-subunit of the α4β1-integrin, preventing leukocyte migration into the central nervous system (9). Pharmacokinetics (PK) and pharmacodynamics of several drugs are frequently altered during pregnancy. However, maternal NAT levels throughout pregnancy and postpartum are still unknown.

Based on findings from the pregnancy in a MS study from 1998, the postpartum period was thought to be associated with an increased risk of relapses (10). Two recently published studies based on contemporary cohorts revealed distinct results with one study observing a return to pre-pregnancy rates and one confirming an increased postpartum relapse rate (4, 11). The return of disease activity following delivery may be reduced by an effective treatment such as NAT (4). However, the use of NAT during lactation period is not recommended due to a lack of safety data. Considering both the benefit of a highly effective disease modifying therapy (DMT) in reducing postpartum relapse risk and the desire to breastfeed with its significant health benefits for both the mother and the infant, some pwMS may choose to breastfeed while continuing or starting NAT therapy. Previous studies, although limited by small sample sizes, observed minimal transfer of NAT into mature breastmilk (12–15).

Recently, it has been postulated that the promising blood-derived biomarker neurofilament light chain (NfL) reflects disease activity during pregnancy in pwMS (16). Upon neuroaxonal damage, the cytoskeletal protein is released into cerebrospinal fluid and blood. Serum (s)NfL levels correlate with disease activity, degree of disability, patient outcome and can also provide important indications for therapeutic response (17–23). Variations of sNfL levels in NAT exposed pregnancies and lactation, which may reflect disease activity as well as treatment response, have not yet been investigated. Additionally, beside the well-established correlation of NfL in cerebrospinal fluid and blood, it is not known whether NfL also diffuses into other body fluids such as breastmilk.

The evaluation of the safety profile regarding the use of high-effective DMT during pregnancy and lactation could support the decision of taking of these drugs, particularly in the case of high disease activity. In this study, we determined NAT concentrations in breast milk of eleven nursing women with relapsing remitting multiple sclerosis (RRMS). Additionally, we aimed to advance our knowledge about pregnancy and postpartum associated variations of NAT's PK and sNfL levels in association to disease activity parameters.

## Methods

### Subjects

We included eleven women diagnosed with RRMS according to the 2010 revisions of the McDonald criteria with a highly active disease course (24). Ten patients received NAT in a standard dose of 300mg prior and throughout pregnancy while one patient started NAT in the second trimester (17th gestational week) due to a clinical confirmed relapse. All patients received NAT every four weeks as approved standard interval dosing (SID), except one woman, who received NAT every four weeks until the 30th gestational week and then switched to an extended interval dosing of six weeks due to patient request. All eleven patients were continuously treated with NAT while breastfeeding. Patients were monitored for the occurrence of clinical and radiological disease activity. Clinical visits were performed every twelve weeks during pregnancy and postpartum period and patients were screened for relapses by a trained and experienced neurologist by history and neurological examination applying the neurostatus expanded disability status scale (EDSS) (25). Relapses were defined as new/worsening of neurologic symptoms persisting ≥ 24 hours in the absence of fever or infection. Two brain MRI scans were performed during postpartum period. The first postpartum MRI scan was compared to the last MRI scan performed prior pregnancy. Radiological disease activity was defined by new and/or enlarging T2-hyperintense lesions in MRI scan. One patient was excluded from analysis of radiological disease activity due to MRI scans at different scanners and protocols with limited comparability.

### Ethical Approval

The immunological sub study was performed according to the Declaration of Helsinki and the study protocol was approved by the Ethics Committee of the Faculty of Medicine of the Dresden University of Technology. All participants provided written informed consent.

### Serum and Breastmilk Samples

Serum samples were collected within the six months prior pregnancy, during each trimester, immediately after delivery and during the first six months after delivery. Samples were obtained immediately before and generally 20 minutes after NAT infusions.

Eight of the eleven women provided at least nine serial breastmilk samples after the first up to tenth maintenance infusions after delivery. Samples were collected immediately before NAT infusion, one day and several days after infusion. The other three patients provided one (n=1), three (n=1) or four (n=1) breastmilk samples.

### Quantification of NAT Concentration Using a HL60 Cell Based Fluorescence-Activated Cell Sorting Assay

Serum and breastmilk samples were frozen at -20°C after collection until final preparation. For analysis of NAT concentrations in breastmilk and serum samples, our previously described HL60 cell based FACS assay (FACS Calibur, BD Bioscience, San Jose, CA, USA) was used (26).

### Evaluation of NfL Levels Using Single Molecule Analysis

Serum and breastmilk samples were stored at -20 until after preparation. NfL levels were determined using a Simoa HD-1 instrument (Quanterix, Lexington, MA, USA) (18, 27). The Advantage NF-Light singleplex Kit was used and samples were prepared as defined in the manufacturer’s instructions (Quartered, Lexington, MA, USA). Sample dilution was calculated and done by the instrument. The mean intra-assay coefficient of variation of duplicates was below 10%.

### Statistical Analysis

Normal distribution of data was visually assessed using quantile-quantile plots and confirmed by Shapiro-Wilk test. Quantitative population characteristics were presented as measures of central tendency (mean, median), followed by standard deviation (SD) or range. For the eight women who provided serial breastmilk samples the trapezoidal rule was used to calculate the area under the breastmilk concentration time curves (AUC_0-Tmax_) for each of these women. The average concentration of NAT in breastmilk (C_AVG_) was calculated by dividing the AUC_0-Tmax_ by the number of days from the first breastmilk sample (T_0_) to the last breastmilk sample (T_max_). Additionally, the maximum concentration (C_MAX_) and the time to the maximum concentration was determined. The absolute average and maximum NAT dose to the infant in a 24-hour period and the relative infant dose (RID) was calculated using a method described by Bennett as well as the assumption that the infant will consume approximately 150ml/kg of breastmilk per day (see **Table 1** for calculations) (28). The RID estimates the infants’ exposure to NAT as a percentage of the maternal dose over a 24-hour period. The actual maternal weight at the time or closely after the first postpartum NAT infusion was assessed for each patient. NAT serum concentration levels are presented as median and range and were analyzed by generalized linear mixed models with gamma distribution and log link function because of the right skewed distribution pattern of the data and timepoint as the fixed effect of the model. Bonferroni correction for pairwise tests was used. P-values < 0.05 were considered as statistically significant. SNfL levels are presented as mean ± SD. The pre-pregnancy sNfL level was defined as individual steady state (SS) level in patients received at least six NAT infusions prior conception (n=9). A significant increase in sNfL was defined as ≥ individual SS level + 2xSD in each patient.

**Table 1:** Calculations used to measure NAT content in breastmilk.

Measure	Calculation
Absolute average NAT dose to the infant in a 24-hour period	Multiply C_AVG_ by 150ml/kg/d
Maximum absolute NAT dose to the infant in a 24-hour period	Multiply C_MAX_ by 150ml/kg/d
Relative infant dose (RID)	Multiply C_AVG_ by 150ml/kg/d of breastmilk and by the maternal weight, then divide by the maternal dose
Maximum RID	Multiply C_MAX_ by 150ml/kg/d of breastmilk and by the maternal weight, then divide by the maternal dose

Statistical analyses were performed using the IBM SPSS software for MAC (version 25.0, IBM Corportation, Armonk, NY) and GraphPad Prism (version 7; GraphPad Software, La Jolla, California).

## Results

### Clinical and Radiological Data

Patient characteristics are reported in **Table 2**. Ten of the eleven women received NAT prior conception with a mean of 20 infusions (SD 5) and continued treatment throughout pregnancy. At the time of conception, patients presented with a mean age of 29.4 years (SD 2.7), a mean EDSS of 1.7 (SD 1.3; median 1.5; range 0-4.5) and a mean disease duration of 4.5 years (SD 3.1). All of them were free of clinical disease activity during gestation. After the initiation of NAT following a relapse in the 17th gestational week in one woman, no further evidence of clinical disease activity was detected. All eleven patients decided to continue NAT treatment while breastfeeding after careful consultation. The first NAT infusion postpartum was administered after a mean of 15.5 days (SD 7.6) after the date of delivery. Patients had received a mean of 27 NAT infusions (SD 5) before lactation period. Two patients received only three NAT infusions following delivery before they were switched to another DMT due to positive John Cunningham Virus serostatus with increased risk for progressive multifocal leukoencephalopathy. In both patients, another second line therapy was started with fingolimod and alemtuzumab after a washout period of two and six months, respectively. 10/11 patients including the patient who switched to treatment with fingolimod were free of clinical disease activity during the first nine months postpartum while the patient who was switched to alemtuzumab presented with a relapse after a six months washout period of NAT.

**Table 2:** Patient demographics and baseline characteristics (N = 11).

Characteristics	Values
Age (years), mean (SD)	29.4 (2.7)
Disease duration (years), mean (SD)	4.5 (3.1)
EDSS, mean (SD)	1.7 (1.3)
EDSS, median (range)	1.5 (1-3)
Number of natalizumab infusions	
prior pregnancy, mean (SD)	20 (15)^1^
before lactation, mean (SD)	27 (17)^2^

EDSS, Expanded Disability Status Scale.

^1^n = 10 patients, ^2^n = 11 patients.

No new and/or enlarging T2 lesions were detected in the first and second postpartum brain MRI scans performed after a median of 1.5 months (range 1 to 3) and a median of 8 months (range 4 to 10), respectively.

### NAT Concentration in Breastmilk

**Figure 1:**
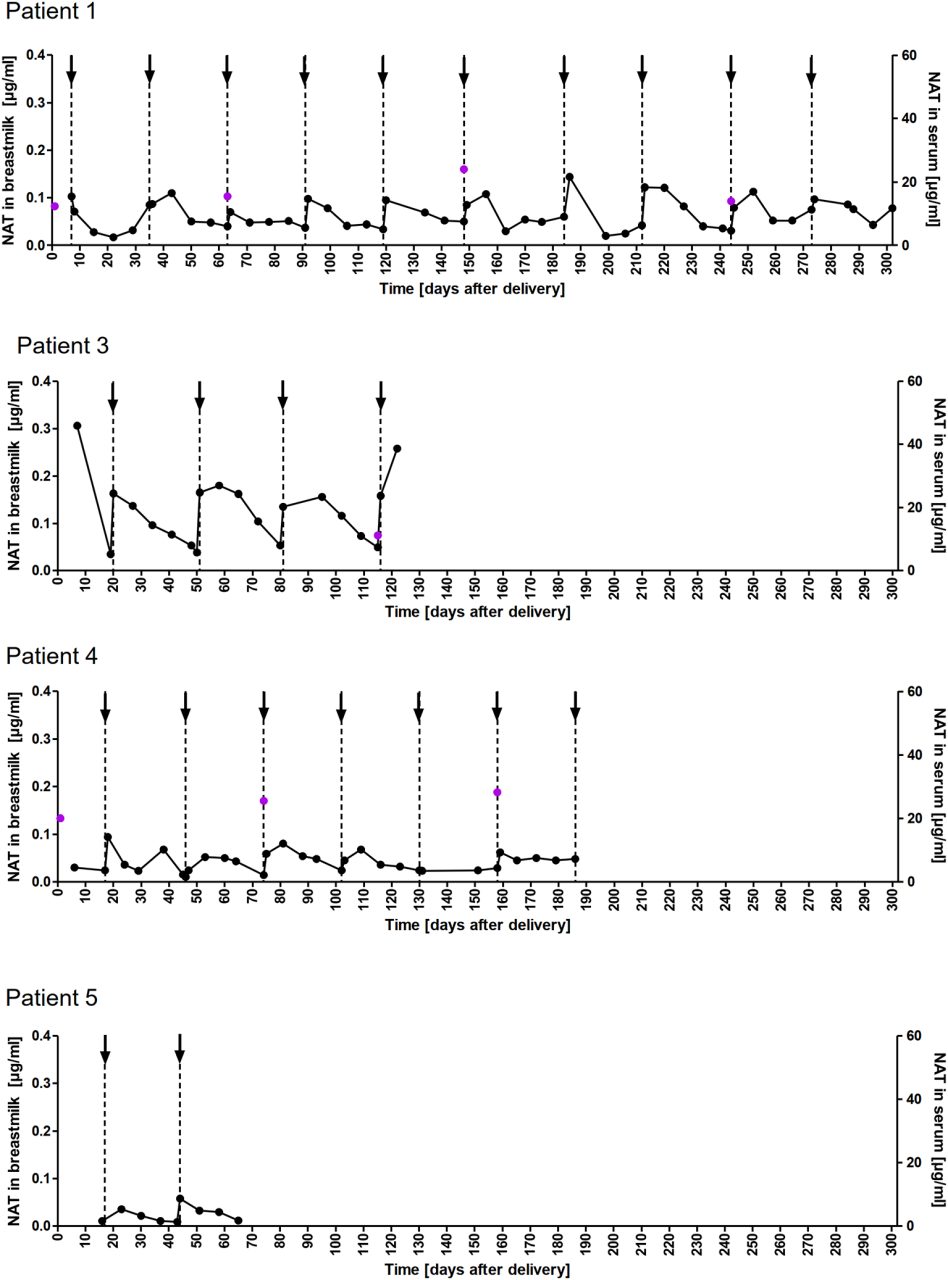
NAT concentration in breastmilk and serum samples of nursing mothers. NAT concentrations (µg/ml) in breastmilk (●) and serum (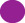) samples of patients 1,3,4 and 5 from delivery (d0) up to 302 days postpartum with up to 10 maintenance NAT infusions (↓).

**Figure 2:**
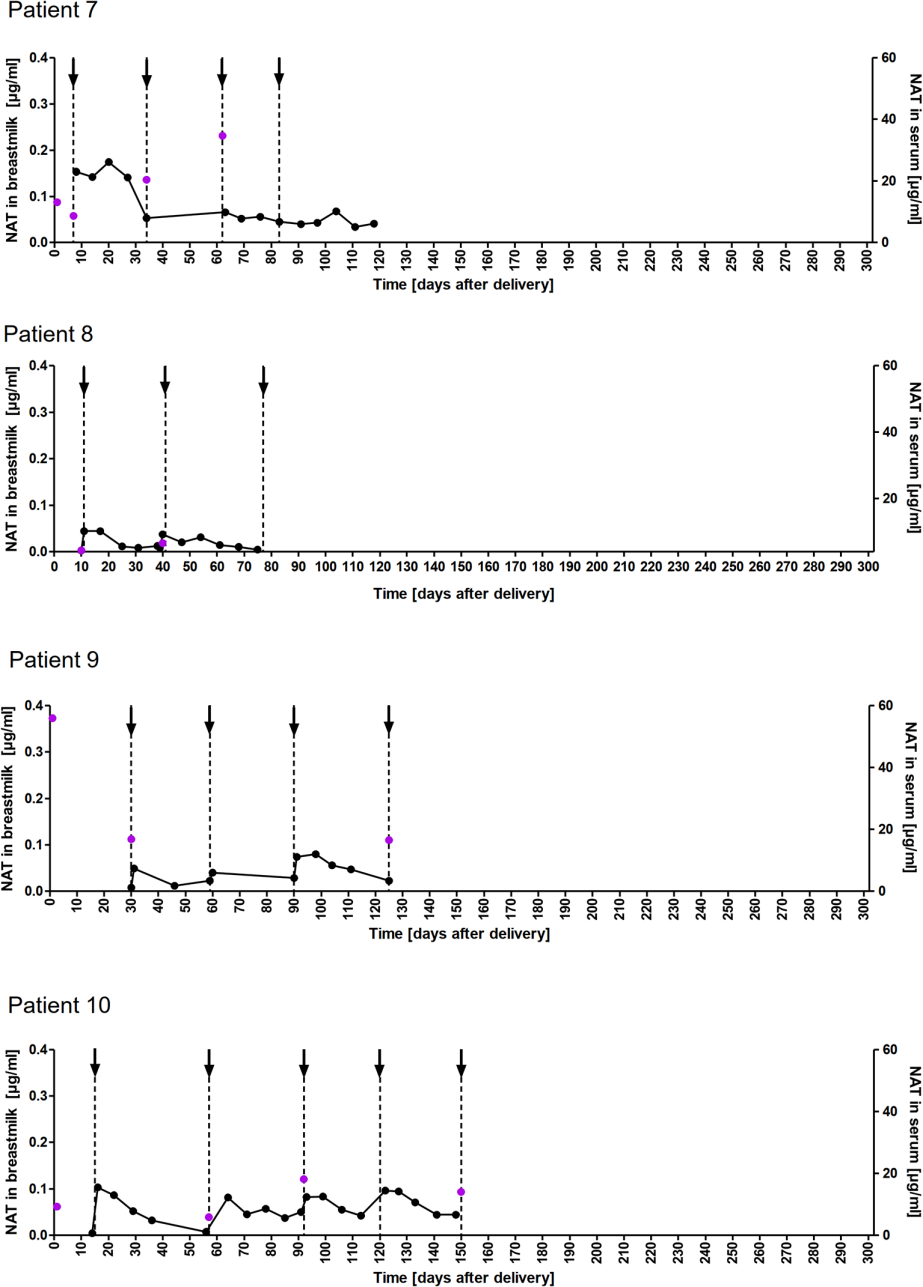
NAT concentration in breastmilk and serum samples of nursing mothers. NAT concentrations (µg/ml) in breastmilk (●) and serum (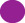) samples of patients 7-10 from delivery (d0) up to 150 days postpartum with up to five maintenance NAT infusions (↓).

A total of 178 breastmilk samples were provided by eleven women, with eight patients donating at least nine serial samples (range 9-51) while the other three patients provided one to four samples, respectively. NAT was detectable in varying concentrations in all breast milk samples with a median concentration of 0.049µg/ml (range 0.002–0.306µg/ml). Pharmacokinetic curves for patients with serial samples are shown in **Figure 1** (patient 1, 3-5) and **Figure 2** (patient 7-10) and PK parameters are detailed in **Table 3**. The mean average NAT concentration was low at 0.06 µg/ml (SD 0.05) in these eight patients.

**Table 3:** Pharmacokinetic parameters of NAT in breastmilk among eight patients with serial samples.

Parameters	Patient 1	Patient 3	Patient 4	Patient 5	Patient 7	Patient 8	Patient 9	Patient 10	Mean (SD)
AUC _0-Tmax_ µg/ml	18.73	14.68	7.39	1.26	8.02	1.34	3.63	7.39	
C_AVG_, µg/ml	0.062	0.120	0.040	0.019	0.068	0.018	0.029	0.050	0.50 (0.034)
C_MAX,_ µg/ml	0.0144	0.306	0.094	0.058	0.174	0.044	0.074	0.103	0.126 (0.085)
Absolute infant dose by C_AVG_, mg/kg/d	0.009	0.018	0.006	0.003	0.010	0.003	0.004	0.007	0.007 (0.005)
Absolute infant dose by C_MAX_, mg/kg/d	0.021	0.046	0.014	0.009	0.026	0.007	0.011	0,015	0.019 (0.013)
RID from C_AVG_, %	0.02	0.10	0.02	0.04	0.06	0.03	0.04	0.04	0.04 (0.03)
RID from C_MAX_, %	0.05	0.3	0.05	0.12	0.14	0.07	0.10	0.07	0.107 (0.069)
Maintenance NAT infusion^1^, n	10	5	7	2	4	2	3	4	4.6 (2.7)
Maternal weight, kg	72	84	64	80	66	64	79	57	71 (10)
Breastmilk, samples, n	51	20	31	9	14	13	11	20	Median (range) 17 (9-51)

AUC, area under the drug concentration time curve; CAVG, average drug concentration across the dose interval; CMAX, maximum drug concentration across the dose interval; RID, relative infant dose; NAT, Natalizumab.

^1^Infusions during collection period of breastmilk samples.

For patient 2, 6 and 11 no calculations due to limited number of samples was performed.

Taken into account that we do not have breastmilk samples from the first hours after NAT infusion, the maximum NAT concentration was measured one to eight days (in one case after 22 days) after infusion with a mean maximum concentration of 0.12 (SD 0.09 µg/ml). Based on C_AVG,_ the mean absolute infant dose of NAT was 0.008mg/kg/d (SD 0.005), and the mean RID based on C_AVG_ was 0.04% (SD 0.03). Corresponding serum samples were available for 7/8 patients with a median NAT concentration of 15.5 µg/ml (range 3.6 to 55.9). The calculated breastmilk to serum ratio from samples obtained immediately before the next NAT infusion, ranged between 1/228 to 1/2100 equivalent to less than 1%.

### NAT Concentration in Serum Prior, During, and After Pregnancy

**Figure 3:**
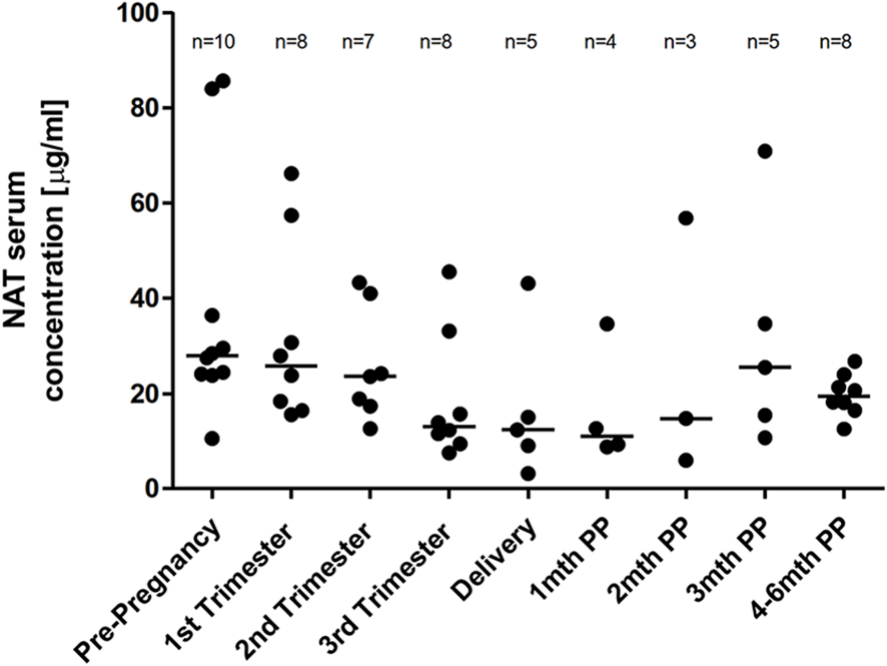
Median NAT serum trough levels across pregnancy. Median NAT trough concentrations (µg/ml) and range in serum samples prior pregnancy, during each trimester and up to six months postpartum. Data were analyzed by general linear mixed models for repeated measures.

A median NAT serum trough (preinfusion) concentration of 27.9 µg/ml (range 10.5 to 85.6) was observed prior pregnancy. Gestation was associated with a non-significant decrease of the median NAT concentration with a percentage difference of 53.1% between levels measured prior pregnancy and in the third trimester. NAT serum trough concentration at month four to six was comparable to NAT concentration prior pregnancy (**Figure 3**). For the median NAT serum concentration measured 20 minutes following infusion, a dynamic similar to NAT trough concentration was observed during pregnancy with a median post infusion concentration of 148 µg/ml (range 74.5 to 286.5) prior pregnancy and 130.5 µg/ml (range 60.5 to 212.5) in the third trimester, respectively (*p* > 0.999).

### SNfL Levels Prior, During, and After Pregnancy

**Figure 4:**
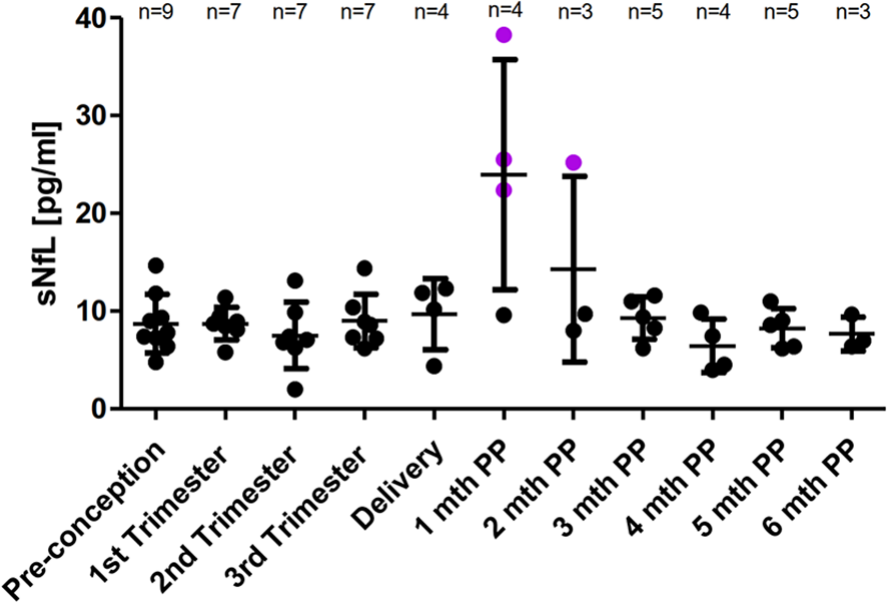
Mean sNfL levels across pregnancy. SNfL mean levels ± SD prior pregnancy, during each trimester and up to six months postpartum. Pre-pregnancy sNfL value in patients having received at least six NAT infusions prior conception was defined as individual steady state value. A relevant increase of sNfL was defined as sNfL value ≥ SS ± 2xSD. A relevant sNfL increase is labeled in purple.

A mean sNfL level of 8.7 pg/ml (SD 3.0, range 4.8 to 14.7pg/ml) was determined pre-pregnancy in nine patients who received at least seven infusions prior conception. During pregnancies, sNfL levels remained at low and stable values in these nine patients. For the patient who received only five infusions prior conception a sNfL concentration of 23.7 pg/ml followed by a constantly decrease during pregnancy was observed. For the early postpartum period, a significant transient peak in the first (n=3) or second (n=1) month following delivery with an up to 6-fold increase to individual SS value (range 22.4-38.3 pg/ml) in four of five patients with available serum samples was revealed. For none of these four patients, clinical nor radiological disease activity in follow up brain MRI scan was documented. During the third and up to the sixth month following delivery low sNfL concentration were observed and linked to a stable disease course (including the one with only five infusions prior conception and the two patients who only received three NAT infusions after delivery) (**Figure 4**).

### NfL in Breastmilk

NfL was detectable in the majority of analyzed breastmilk samples (158 of 163) with a median concentration of 1.7 pg/ml (range 0.004 to 18.1). A correlation with NfL levels in serum was not observed.

## Discussion

In this study of NAT-treated highly active RRMS patients, therapeutic drug monitoring revealed a low transfer of NAT into breastmilk and a small, non-significant decrease of NAT serum trough concentration across pregnancy. Although no evidence of clinical and radiological disease activity was detected in NAT exposed pregnancies and postpartum period up to six months, puerperium was linked to a transient sNfL peak in some patients.

While IgA represents the primary Ig in breastmilk, IgG based monoclonal antibodies (mAbs) are largely precluded from transfer into breastmilk due to large molecule size and limited transport mechanisms (29). For the determination of drug transfer into human breastmilk, the IgG subclass also appears to be important, as there is more IgG4 than IgG1 in mature breastmilk (30). The majority of mAbs used for different medical conditions are of the IgG1 subclass whereas NAT is an IgG4 subclass, which implicates a potential greater transfer of NAT into breastmilk (30). However, in this study, we observed very low transfer of NAT into breastmilk similar to previous reports (12–15). The average and maximum concentration of NAT in mothers´ milk were low in all patients with serial samples and tended to peak within the first eight days, although maximum levels could occur up to 22 days from infusion. Although the analysis of the PK of NAT in breastmilk during the first 24 hours after infusion was not part of our study, previous data suggest that the time to peak concentration will be at minimum 24 hours (12, 14, 15). However, these previous findings were obtained from smaller sample size. The median RID estimated by C_AVG_ was 0.04% and the median RID estimated by C_MAX_ was 0.11%, which is in line with recently published data and well below the 10% that has generally been considered safe for breastfeeding an infant (15, 31). The amount of NAT reaching the breastmilk is likely degraded in the infant´s gastrointestinal tract (32). However, the neonatal Fc receptor (nFcR) may allow passage of some indigested IgG molecules into the circulation, although it may be stripped from the IgG during passage through the gut (33). NAT was not detectable in serum of two infants during exposed breastfeeding as previously reported, supporting the assumption that there is no substantial transfer of mAbs from breastmilk *via* nFcR (15). In recent studies, an accumulation of NAT in breastmilk over time with a consecutive increase of RID up to 5.3% was revealed in individual patients (12, 13). Here, we could not observe a relevant time-dependent accumulation in a significant group of eight patients with serial samples up to 302 days after delivery and up to ten maintenance infusions, which is in line with previous data (15). However, due to the limited data available so far and inter-individual variability of NAT levels, an accumulation of NAT in breastmilk in individual patients cannot be ruled out. During NAT exposed lactation no adverse effects on their infants or breastfeeding difficulties were reported by the patients of our study. Overall, the low RID observed in this study indicate a minimal drug exposure of the infant through breastmilk alone.

Pregnancy is known as a state where PK changes are more pronounced and more rapid than during any other period of life with potential effects on any level of the disposition of drugs. Given the importance to maintaining an adequate treatment response during pregnancy in highly active MS, it is mandatory to understand whether pregnancy affects drug levels. For NAT, median serum trough concentration measured prior to pregnancy was similar to those levels reported in previous studies (26, 34–37). Although pregnancy was associated with a non-significant drop of median NAT serum trough levels, median NAT trough concentration at the third trimester was still within the previous described range of pre-infusion NAT levels (26, 34–36). Serum NAT concentration is known to correspond with the percentage of α4-integrin receptor saturation with an adequate saturation estimated as et al ≥ 70%-80%. Above a threshold of 2µg/ml, α4-integrin receptor saturation will roughly fall between 70% and 100% (34). Thus, NAT serum levels in the third trimester are likely beyond what is necessary to maintain α4-integrin receptor saturation. Although pregnancy is associated with an increase of the plasma volume by around 40-50%, with their limited volume of distribution, the PKs of mAbs are not considered likely to be substantially altered (38). However, the current findings suggest that there may be pregnancy-related changes that marginally increase NAT clearance. The small drop of NAT level during pregnancy may not be clinically significant and measurement of serum concentration across pregnancy seems not to be required.

Disease reactivation following NAT cessation prior to or during pregnancy is well recognized and can lead to the accumulation of permanent disability (6, 39–41). Even the postpartum period entails an increased relapse risk and could be linked to a high level of inflammatory radiographic disease activity as it was shown in a recent study (3, 4, 11, 42). None of our patients who received NAT prior to, across and after pregnancy or who started treatment due to a relapse in the second trimester suffered from a (further) relapse during gestation or in the first six months postpartum. In two patients with increased risk for progressive multifocal leukoencephalopathy, NAT therapy was withdrawn after the third postpartum infusion. With attention to the potential recurrence of disease activity, which typically peaks four to seven months following NAT discontinuation, in both patients another DMT was initiated with fingolimod and alemtuzumab after a two and six months washout period, respectively (43). No evidence for radiological disease activity was revealed in both the first and the second post-partum brain MRI scan, performed at a median of 1.5 and eight months, respectively. Drawn together, NAT was able to prevent clinical and radiological disease activity during pregnancy and in the first six months postpartum in our small patient cohort.

Beside clinical and radiological disease activity parameters, blood derived biomarkers gain more and more attention. The implementation of a blood derived and easy to measure biomarker would be a valuable adjunct for monitoring disease activity during pregnancy in women with MS. Here, we describe pregnancy and postpartum associated changes in sNfL levels in women exposed to NAT. We observed low pre-pregnancy sNfL values in nine patients who received a least seven NAT infusions while a sNfL concentration of 23.7 pg/ml was detected in a patient who received only five infusions prior pregnancy. sNfL levels remained low and stable in the nine patients and decreased in the patient with only five infusions. We revealed a short lasting peak in sNfL with a significant increase up to 6-fold in puerperium in 4/5 patients with available serum samples from early postpartum period. This transient peak was not accompanied by clinical or radiological disease activity. For the further postpartum period from three to six months, low sNfL levels were detected, which were linked to a stable disease course in all patients.

In this study, patients were only monitored through cerebral MRI as spinal cord MRI was not performed regularly. Other factors such as pre-eclampsia, general anesthesia during delivery, trauma, stroke, metabolic diseases, which could be associated with the postpartum sNfL increase, were not reported. For patients treated with alemtuzumab, similar findings about transient sNfL peaks postpartum were reported (23). A recently published study revealed a postpartum increase of sNfL in 49 of 56 patients at risk of developing preeclampsia and speculated that the increase is triggered by parturition per se due to a negative impact on neuronal integrity. However, it was mentioned, that the peripheral nervous system or other tissues might also contribute to the increase in sNfL (44). So far, it remains elusive whether the short lasting increase in sNfL reflects silent disease activity of MS or whether it may be triggered by the stress associated with parturition. Here, we investigated the presence of NfL in breastmilk which was measurable in the majority of samples. According to the small molecule size of NfL of about 60-70kDa, it is not surprising that NfL diffuse into breast milk. However, we could not reveal a serum/breastmilk correlation of NfL which may be caused by the limited number of paired serum/breastmilk samples.

In accordance with previous study results, we can confirm that NAT is transferred into human breastmilk in reassuringly low amounts. The observed alteration of drug levels during pregnancy are small and unlikely to be of clinically significance regarding efficacy. Data presented here suggest a therapeutic benefit for women with highly active RRMS from treatment with NAT during pregnancy and lactation and highlight sNfL as a promising add-on biomarker for monitoring disease activity and therapeutic response in pregnancy and postpartum period.

While rheumatology and gastroenterology societies have already provided guidelines about the use of mAbs during lactation, such guidelines are lacking for neurologic conditions (45, 46). The further acquisition of more data regarding the safety of mAbs during pregnancy and breastfeeding in women with MS, including actual drug exposure and effects on developing infants, clearly needs scientific attention. Further studies are needed to investigate the impact of parturition per se and subclinical MS disease activity on maternal neuronal integrity.

## Data Availability Statement

The raw data supporting the conclusions of this article will be made available by the authors, without undue reservation.

## Ethics Statement

The studies involving human participants were reviewed and approved by Ethics Committee of the Faculty of Medicine of the Dresden University of Technology. The patients/participants provided their written informed consent to participate in this study.

## Author Contributions

Study concept and design: UP, KA, and TZ. Acquisition of data: UP. Analysis and interpretation of data: UP. Drafting of the manuscript: UP, KA, and TZ. Critical revision of the manuscript for important intellectual content: RH and HI. Statistical analysis: UP and RH. Study supervision: KA and TZ. All authors contributed to the article and approved the submitted version.

## Conflict of Interest

UP received speaker fee from Merck, Biogen and Bayer and personal compensation from Biogen and Roche for consulting service. RH has received travel compensation from Celgene and Sanofi. HI received speaker fee from Roche. KA received personal compensation from Roche, Sanofi, Alexion, Teva, Biogen and Celegene for consulting service. TZ reports consulting or serving at speaker bureaus for Biogen, Celgene, Roche, Novartis, Celgene, Merck and Sanofi as well as research support from Biogen, Novartis, Merck and Sanofi.

## Publisher’s Note

All claims expressed in this article are solely those of the authors and do not necessarily represent those of their affiliated organizations, or those of the publisher, the editors and the reviewers. Any product that may be evaluated in this article, or claim that may be made by its manufacturer, is not guaranteed or endorsed by the publisher.

## Abbreviations

AUC, area under the curve; C_AVG_, average concentration; C_MAX_, maximum concentration; DMT, disease modifying therapy; EDSS, expanded disability status scale; FACS, fluorescence activated cell sorting; Ig, immunoglobulin; mAbs, monoclonal antibodies; MS, multiple sclerosis; NAT, natalizumab; PK, pharmacokinetic; pwMS, people with multiple sclerosis; RID, relative infant dose; RRMS, relapsing remitting multiple sclerosis; SD, standard deviation; SID, standard interval dosing; SIMOA, single molecule array technology; (s)NfL, (serum) neurofilament light chain; SS, steady state.

## References

[1] Nicot AB. Gender and Sex Hormones in Multiple Sclerosis Pathology and Therapy. Front Biosci (Landmark Ed) (2009) 14:4477. 10.2741/3543 19273365 PMC2689399

[2] Harbo HF Gold R Tintore M. Sex and Gender Issues in Multiple Sclerosis. Ther Adv Neurol Disord (2013) 6:237–48. 10.1177/1756285613488434 PMC3707353 23858327

[3] Confavreux C Hutchinson MD Hours MM Cortinovis-Tourniaire P Moreau T. The Pregnancy in Multiple Sclerosis Group. Rate of Pregnancy-Related Relapse in Multiple Sclerosis. N Eng J Med (1998) 339:285–91. 10.1056/NEJM199807303390501 9682040

[4] Yeh WZ Widyastuti PA van der Walt A Stankovich J Havrdova E Horakova D. Natalizumab, Fingolimod and Dimethyl Fumarate Use and Pregnancy-Related Relapse and Disability in Women With Multiple Sclerosis. Neurology (2021) 96(24):e2989–e3002. 10.1212/WNL.0000000000012084 PMC8253565 33879599

[5] Sempere AP Berenguer.Ruiz L Feliu-Rey E. Rebound of Disease Activity During Pregnancy After Withdrawal of Fingolimod. Eur J Neurol (2013) 20(8):e109–10. 10.1111/ene.12195 23829238

[6] De Giglio L Gasperini C Tortorella C Trojano M Pozzilli C. Natalizumab Discontinuation and Disease Restart in Pregnancy: A Case Series. Acta Neurol Scand (2015) 131:336–40. 10.1111/ane.12364 25598313

[7] Martinelli V Colombo B Costa GD Libera DD Moiola L Falini A. Recurrent Disease-Activity Rebound in a Patient With Multiple Sclerosis After Natalizumab Discontinuations for Pregnancy Planning. Mult Scler J (2013) 22:1506–8. 10.1177/1352458513492246 23773984

[8] Summary of Product Characteristics of Tysabri (natalizumab). Available at: https://www.ema.europa.eu/en/documents/product-information/tysabri-epar-product-information_en.pdf. [Accessed March 01, 2021].

[9] Polman CH O'Connor PW Havrdova E Hutchinson M Kappos L Miller DH. A Randomized, Placebo-Controlled Trial of Natalizumab for Relapsing Multiple Sclerosis. N Eng J Med (2006) 354(9):899–910. 10.1056/NEJMoa044397 16510744

[10] Confavreux C Hutchinson M Hours MM Cortinovis Tourniaire P Moreau T. Rate of Pregnancy-Related Relapse in Multiple Sclerosis. N Engl J Med (1998) 339:285–91. 10.1056/NEJM199807303390501 9682040

[11] Langer-Gould A Smith J Albers K Wu J Kerezsi E McClearnen K. Pregnancy-Related Relapses and Breastfeeding in a Contemporay Multiple Sclerosis Cohort. Neurology (2020) 94(18):e1939–e49. 10.1212/WNL.0000000000009374 PMC7274922 32284359

[12] Baker TE Cooper SD Kessler L Hale TW. Transfer of Natalizumab Into Breast Milk in a Mother With Multiple Sclerosis. J Hum Lact (2015) 31:233–6. 10.1177/0890334414566237 25586712

[13] Proschmann U Thomas K Thiel S Hellwig K Ziemssen T. Natalizumab During Pregnancy and Lactation. Mult Scler J (2017) 24:1627–34. 10.1177/1352458517728813 28857686

[14] Matro R Martin CF Wolf D Shah SA Mahadevan U. Exposure Concentrations of Infants Breastfed by Women Receiving Biologic Therapies for Inflammatory Bowel Diseases and Effects of Breastfeeding on Infections and Development. Gastroenterology (2018) 155:696–704. 10.1053/j.gastro.2018.05.040 29857090

[15] Ciplea AI Langer-Gould A de Vries A Schaap T Thiel S Ringelstein M. Monoclonal Antibody Treatment During Pregnancy and/or Lactation in Women With MS or Neuromyelitis Optica Spectrum Disorder. Neurol Neuroimmunol Neuroinflamm (2020) 7:e723. 10.1212/NXI.0000000000000723 32327455 PMC7188475

[16] Cuello JP Ginés MLM Kuhle J Domínguez JMG Ros AL Delgado FR. Neurofilament Light Chain Levels in Pregnant Multiple Sclerosis Patients: A Prospective Cohort Study. Eur J Neurol (2019) 26(9):1200–4. 10.1111/ene.13965 30977955

[17] Khalil M Teunissen CE Otto M Piehl F Sormani MP Gattringer T. Neurofilaments as Biomarkers in Neurological Disorders. Nat Rev Neurol (2018) 14:577–89. 10.1038/s41582-018-0058-z 30171200

[18] Disanto G Barro C Benkert P Naegelin Y Schädelin S Giardiello A. Serum Neurofilament Light: A Biomarker of Neuronal Damage in Multiple Sclerosis. Ann Neurol (2017) 81:857–70. 10.1002/ana.24954 PMC5519945 28512753

[19] Kuhle J Barro C Disanto G Mathias A Soneson C Bonnier G. Serum Neurofilament Light Chain in Early Relapsing Remitting MS Is Increased and Correlates With CSF Levels and With MRI Measures of Disease Severity. Mult Scler J (2016) 22:1550–9. 10.1177/1352458515623365 26754800

[20] Kuhle J Nourbakhsh B Grant D Morant S Barro C Yaldizli Ö. Serum Neurofilament Is Associated With Progression of Brain Atrophy and Disability in Early MS. Neurology (2017) 88:826–31. 10.1212/WNL.0000000000003653 PMC5331872 28148632

[21] Kuhle J Plavina T Barro C Disanto G Sangurdekar D Singh CM. Neurofilament Light Levels Are Associated With Long-Term Outcomes in Multiple Sclerosis. Mult Scler J (2019) 92(10):e1007–e15. 10.1177/1352458519885613 PMC7604552 31680621

[22] Kuhle J Kropshofer H Haering DA Kundu U Meinert R Barro C. Blood Neurofilament Light Chain as a Biomarker of MS Disease Activity and Treatment Response. Neurology (2019) 92(10):e1007–e15. 10.1212/WNL.0000000000007032 PMC6442011 30737333

[23] Akgün K Kretschmann N Haase R Proschmann U Kitzler HH Reichmann H. Profiling Individual Clinical Responses by High-Frequency Serum Neurofilament Assessment in MS. Neurol Neuroimmunol Neuroinflamm (2019) 6:e555. 10.1212/NXI.0000000000000555 31119188 PMC6501638

[24] Polman CH Reingold SC Banwell B Clanet M Cohen JA Filippi M. Diagnostic Criteria for Multiple Sclerosis: 2010 Revisions to the McDonald Criteria. Ann Neurol (2011) 69:292–302. 10.1002/ana.22366 21387374 PMC3084507

[25] Inojosa H Proschmann U Akgün K Ziemssen T. Should We Use Clinical Tools to Identify Disease Progression? Front Neurol (2021) 11:628542. 10.3389/fneur.2020.628542 33551982 PMC7859270

[26] Sehr T Proschmann U Thomas K Marggraf M Straube E Reichmann H. New Insights Into the Pharmacokinetics and Pharmacodynamics of Natalizumab Treatment for Patients With Multiple Sclerosis, Obtained From Clinical and *In Vitro* Studies. J Neuroinflamm (2016) 13:16. 10.1186/s12974-016-0635-2 PMC4924246 27349895

[27] Wilson DH Rissin DM Kan CW Fournier DR Piech T Campbell TG. The Simoa HD-1 Analyzer: A Novel Fully Automated Digital Immunoassay Analyzer With Single-Molecule Sensitivity and Multiplexing. J Lab Autom (2015) 21:533–47. 10.1177/2211068215589580 26077162

[28] Bennett P Astrup-Jensen A Bates CJ Begg EJ Edwards S Lazarus C Matheson I. Drugs and Human Lactation. Amsterdam, Elsevier (1996).

[29] Butler J Kehrli M. Immunoglobulins and Immunocytes in the Mammary Gland and Its Secretions. In: Mucosal Immunology (2004). Amsterdam: Elsevier (2005). p. 1763–93. 10.1016/B978-012491543-5/50107-8

[30] Rodríguez-Camejo C Puyol A Fazio L Rodríguez A Villamil E Andina E. Antibody Profile of Colostrum and the Effect of Processing in Human Milk Banks: Implications in Immunoregulatory Properties. J Hum Lact (2017) 34:137–47. 10.1177/0890334417706359 28586632

[31] Newton ER Hale TW. Drugs in Breast Milk. Clin Obstet Gynecol (2015) 58:868–84. 10.1097/GRF.0000000000000142 26457856

[32] Hurley WL Theil PK. Perspectives on Immunoglobulins in Colostrum and Milk. Nutrients (2011) 3:442–74. 10.3390/nu3040442 PMC3257684 22254105

[33] Giragossian C Clark T Piche-Nicholas N J Bowman C. Neonatal Fc Receptor and Its Role in the Absorption, Distribution, Metabolism and Excretion of Immunoglobulin G-Based Biotherapeutics. Curr Drug Metab (2013) 14:764–90. 10.2174/13892002113149990099 23952252

[34] van Kempen ZL Leurs CE Witte BI de Vries A Wattjes MP Rispens T. The Majority of Natalizumab-Treated MS Patients Have High Natalizumab Concentrations at Time of Re-Dosing. Mult Scler J (2017) 24:805–10. 10.1177/1352458517708464 PMC5971363 28485678

[35] Plavina T Muralidharan KK Kuesters G Mikol D Evans K Subramanyam M. Reversibility of the Effects of Natalizumab on Peripheral Immune Cell Dynamics in MS Patients. Neurology (2017) 89:1584–93. 10.1212/WNL.0000000000004485 PMC5634662 28916537

[36] van Kempen ZLE Hoogervorst ELJ Wattjes MP Kalkers NF Mostert JP Lissenberg-Witte BI. Personalized Extended Interval Dosing of Natalizumab in MS: A Prospective Multicenter Trial. Neurology (2020) 95:e745–54. 10.1212/WNL.0000000000009995 32690785

[37] Proschmann U Inojosa H Akgün K Ziemssen T. Natalizumab Pharmacokinetics and-Dynamics and Serum Neurofilament in Patients With Multiple Sclerosis. Front Neurol (2021) 12:650530. 10.3389/fneur.2021.650530 33935948 PMC8079654

[38] Stone RH Hong J Jeong H. Pharmacokinetics of Monoclonal Antibodies Used for Inflammatory Bowel Diseases in Pregnant Women. J Clin Toxicol (2014) 4(4):209. 10.4172/2161-0495.1000209 26539323 PMC4629634

[39] Portaccio E Annovazzi P Ghezzi A Zaffaroni M Moiola L Martinelli V. Pregnancy Decision-Making in Women With Multiple Sclerosis Treated With Natalizumab: I: Fetal Risks. Neurology (2018) 90:e823–31. 10.1212/WNL.0000000000005067 29438046

[40] Kleerekooper I van Kempen ZLE Leurs CE Dekker I Rispens T Lissenberg-Witte BI. Disease Activity Following Pregnancy-Related Discontinuation of Natalizumab in MS. Neurol Neuroimmunol Neuroinflamm (2018) 5(1):e424. 10.1212/NXI.0000000000000424 29379823 PMC5778770

[41] Razaz N Piehl F Frisell T Langer-Gould AM McKay KA Fink K. Disease Activity in Pregnancy and Postpartum in Women With MS Who Suspended Rituximab and Natalizumab. Neurol Neuroimmunol Neuroinflamm (2020) 7(6):e903. 10.1212/NXI.0000000000000903 33087582 PMC7641107

[42] Houtchens M Bove R Healy B Houtchens S Kaplan TB Mahlanza T. MRI Activity in MS and Completed Pregnancy: Data From a Tertiary Academic Center. Neurol Neuroimmunol Neuroinflamm (2020) 7(6):e890. 10.1212/NXI.0000000000000890 32917773 PMC7643615

[43] Prosperini L Kinkel RP Miravalle AA Iaffaldano P Fantaccini S. Post-Natalizumab Disease Reactivation in Multiple Sclerosis: Systematic Review and Meta-Analysis. Ther Adv Neurol Disord (2019) 12:175628641983780. 10.1177/1756286419837809 PMC6444403 30956686

[44] Evers KS Huhn EA Fouzas S Barro C Kuhle J Fisch U. Impact of Parturition on Maternal Cardiovascular and Neuronal Integrity in a High Risk Cohort – A Prospective Cohort Study. BMC Pregnancy Childbirth (2019) 19:1–8. 10.1186/s12884-019-2570-6 31690271 PMC6833198

[45] Mahadevan U Robinson C Bernasko N Boland B Chambers C Dubinsky M. Inflammatory Bowel Disease in Pregnancy Clinical Care Pathway: A Report From the American Gastroenterological Association IBD Parenthood Project Working Group. Inflamm Bowel Dis (2019) 25:627–41. 10.1093/ibd/izz037 30821832

[46] Puchner A Gröchenig HP Sautner J Helmy-Bader Y Juch H Reinisch S. Immunosuppressives and Biologics During Pregnancy and Lactation. Wien Klin Wochenschr (2019) 131:29–44. 10.1007/s00508-019-1448-y 30643992 PMC6342891

